# An AI-enabled research support tool for the classification system of COVID-19

**DOI:** 10.3389/fpubh.2023.1124998

**Published:** 2023-03-03

**Authors:** Arti Tiwari, Kamanasish Bhattacharjee, Millie Pant, Shilpa Srivastava, Vaclav Snasel

**Affiliations:** ^1^Department of Applied Mathematics and Scientific Computing, Indian Institute of Technology Roorkee, Roorkee, Uttarakhand, India; ^2^Machine Intelligence in Medicine and Imaging (MI-2) Lab, Mayo Clinic, Phoenix, AZ, United States; ^3^Mehta Family School for Data Science and Artificial Intelligence, Indian Institute of Technology Roorkee, Roorkee, Uttarakhand, India; ^4^CHRIST (Deemed to be University) Delhi NCR, Ghaziabad, India; ^5^Department of Computer Science, VŠB-Technical University of Ostrava, Ostrava, Czechia

**Keywords:** COVID-19, long short-term memory, classification, bi-directional LSTM, Artificial Intelligence

## Abstract

The outbreak of COVID-19, a little more than 2 years ago, drastically affected all segments of society throughout the world. While at one end, the microbiologists, virologists, and medical practitioners were trying to find the cure for the infection; the Governments were laying emphasis on precautionary measures like lockdowns to lower the spread of the virus. This pandemic is perhaps also the first one of its kind in history that has research articles in all possible areas as like: medicine, sociology, psychology, supply chain management, mathematical modeling, etc. A lot of work is still continuing in this area, which is very important also for better preparedness if such a situation arises in future. The objective of the present study is to build a research support tool that will help the researchers swiftly identify the relevant literature on a specific field or topic regarding COVID-19 through a hierarchical classification system. The three main tasks done during this study are data preparation, data annotation and text data classification through bi-directional long short-term memory (bi-LSTM).

## 1. Introduction

Early in the year 2020, the outbreak of COVID-19 created havoc around the world, leading to mental trauma, shattered economies and, above all, the loss of human life. While the researchers and scientists were trying to understand more about the virus and a possible antidote/vaccine for it, the challenge for the Government was to keep its people safe by enforcing preventive measures like lockdowns. The uncertainty of the situation affected almost all sections of society. Despite all this grimness, the scientific and research community was doing its bit through experiments and observations and publishing research articles and reports on its basis. The COVID pandemic, perhaps, also is the first case of its kind that provoked research in all possible dimensions. Although the situation is not alarming anymore, with people getting vaccinated and economies getting back on pace, the research on COVID-19 is still continuing, and a noticeable quantity of research articles are being published.

The internet now contains a plethora of literature dedicated to the various aspects of COVID-19 ranging from studies related to lab experiments to clinical studies to vaccines and drug development to diagnostic techniques and many more. There are several studies dedicated to economics and mathematical models, forecasting methods to estimate the spread of the virus, supply chain models and several others.

### 1.1. Bibliometric analysis

A selected bibliometric analysis was performed on the CORD-19 dataset for articles related to COVID-19 which were later used for model training and database development. The results are obtained to show the trend of publications for COVID-19 articles and the “*terms*” used in the paper to label the classes.

**Figure 1:**
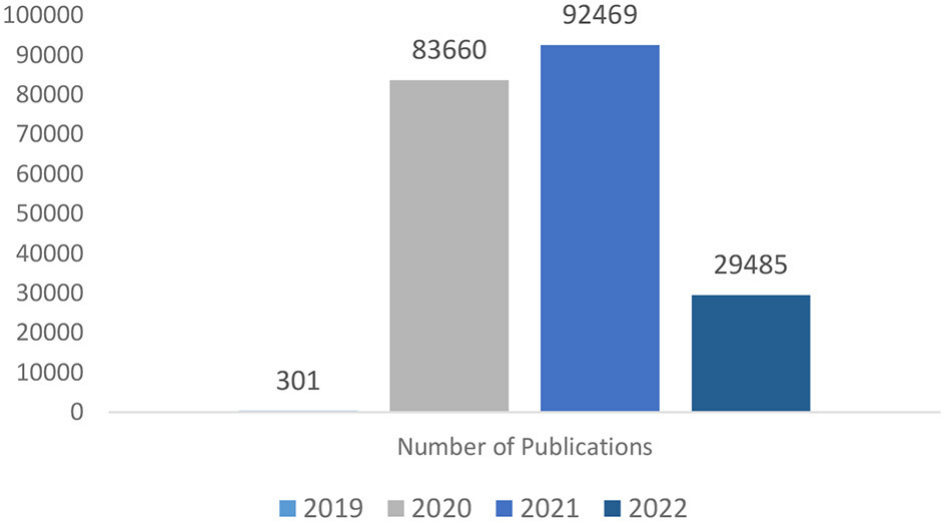
Year-wise number of publications listed in CORD-19 dataset.

Figure 1 shows that in 2019, at the onset of COVID-19, the publications were 301 in number, which raised to 83,660 in 2020 and further raised to 92,469 in 2021 and although in 2022, the number of articles became 29,485, the trends are good enough to indicate that the research is still continuing in this area with new research papers being published from time to time.

**Figure 2:**
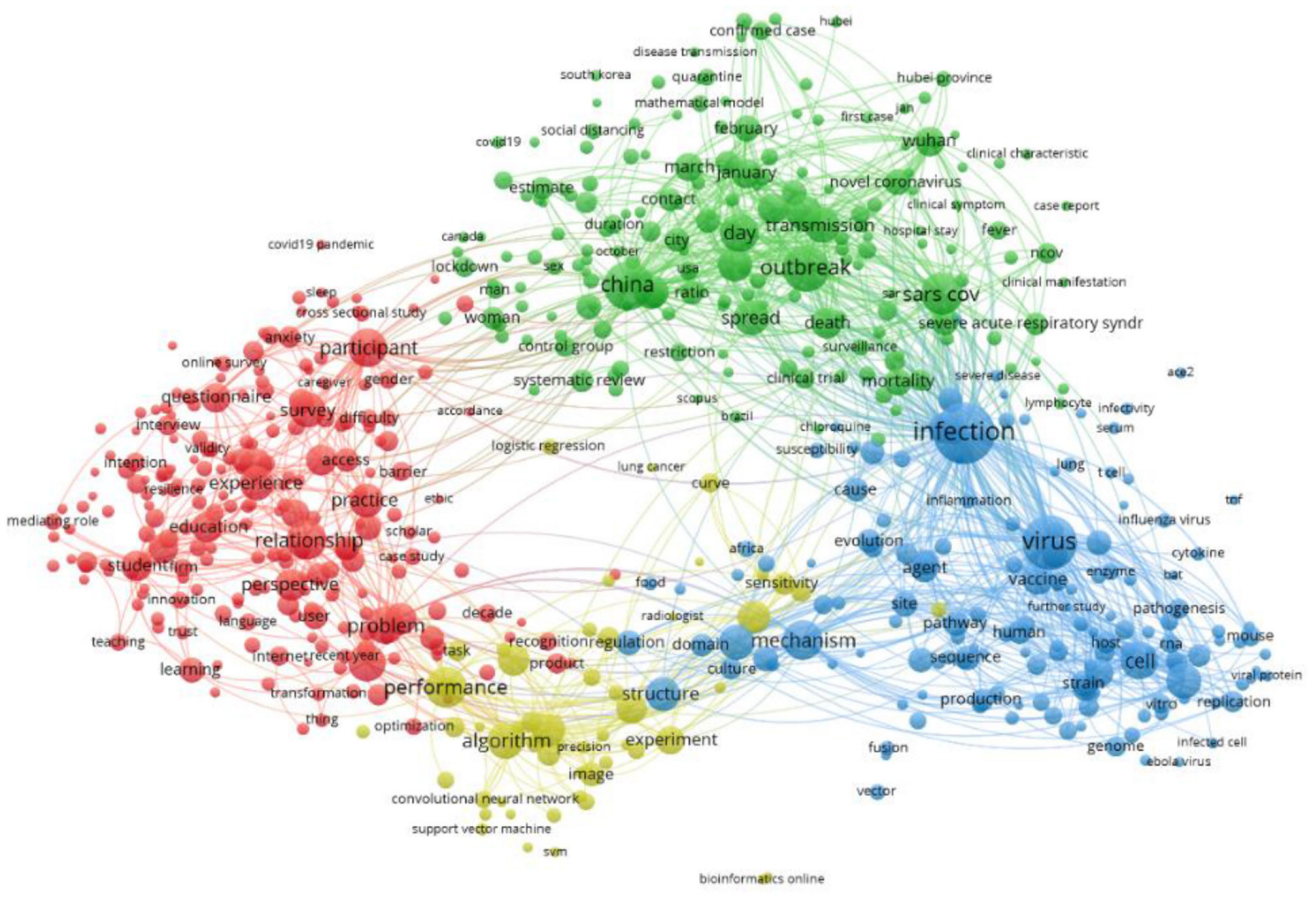
Network visualization map based on Abstract and Title.

Figure 2 shows a network visualization created using Vosviewer (https://www.vosviewer.com/). The network map includes the terms/items (object of interest) represented by a circle driven by the title and abstract of the selected articles and the links between the terms based on their pair-wise occurrence. The higher the occurrence of an item, the bigger the circle. In this map total of 612 terms are selected and grouped into four non-overlapping clusters. Cluster-one (red) consists of 223 terms, cluster-two (green) contains 186 items, cluster-three (blue) incorporate 149 items, and cluster-four (yellow) contains 54 terms.

In Figure 2, the term “infection” is depicted with the biggest circle, as this term shares the highest co-occurrence with the other terms.

**Figure 3:**
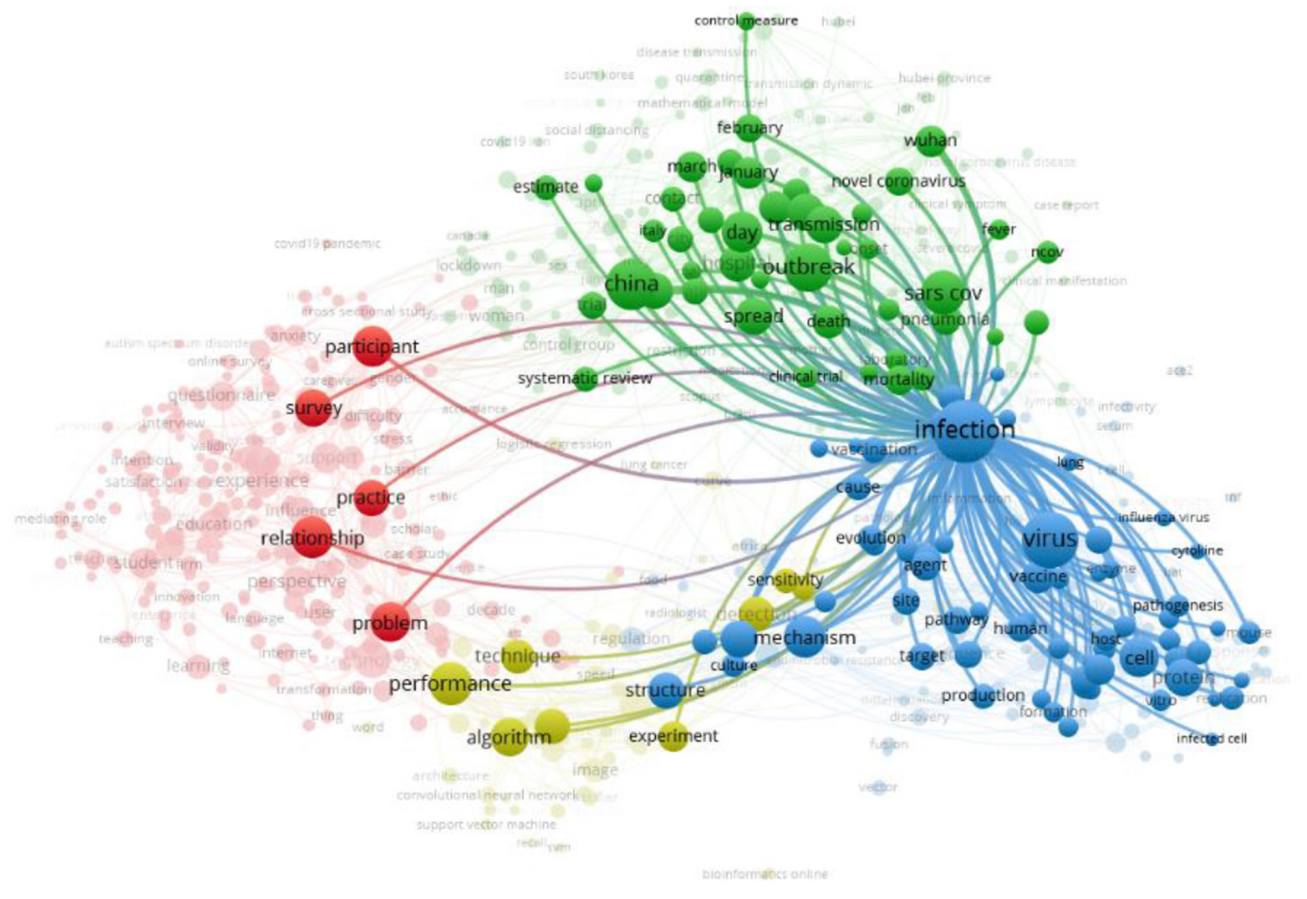
Network visualization map of the term “infection”.

Figure 3 describes the network visualization map of the term/item “infection,” which possesses the highest occurrence value and link strength value as 433 and 5,014, respectively. The link strength value shows the number of articles where two terms occurred together.

**Figure 4:**
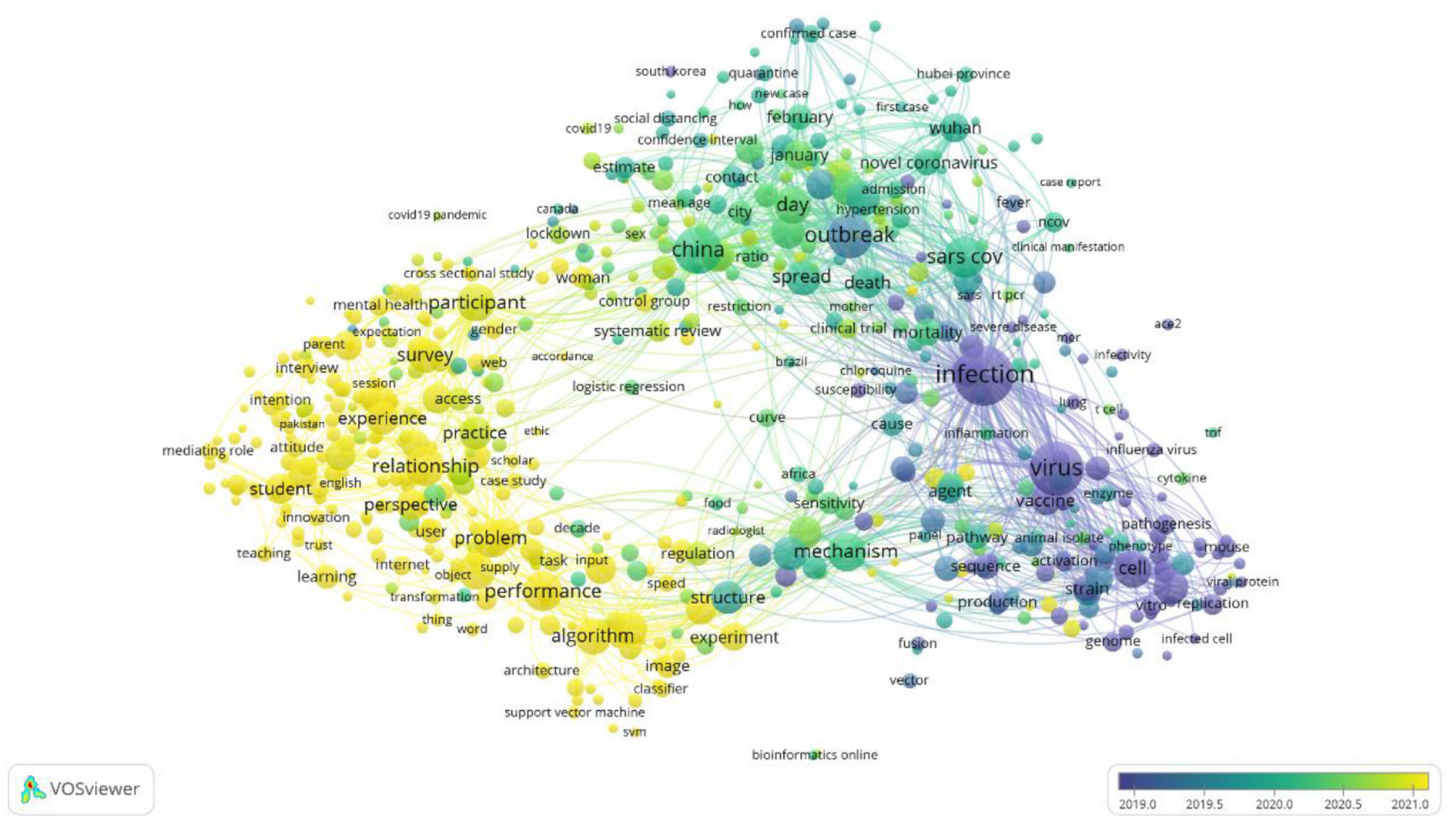
Overley visualization of the articles based on publication year.

On the basis of the publication years of the selected articles, an overlay visualization map is created in Figure 4. This visualization of this map is identical to the network map, however, its interpretation is based on the score of the average publication year.

This map shows the focus shifts on the area of research publication throughout the year 2019 to the year 2021. Since early to mid-2019, the published articles were subjected toward the infection, virus, and vaccine. From mid-2019 to mid-2020 the published articles were tend toward China, the outbreak, SARS-CoV, and its spread. After mid-2020, the articles are focused on problem-solving, algorithms, perspective, experiments and performance.

### 1.2. Need for a dedicated research support tool

The graphs given above clearly indicate, how the research is growing in the area of COVID-19. These graphs also show that there are several categories (fields) of research and every category can be further divided into sub-categories (subfields). For a new researcher, digging into this plethora of information can be quite overwhelming. It becomes difficult for a researcher to identify the correct literature relevant to one's area of interest. This difficulty may be eased to some extent if there is a dedicated platform which can easily guide them to their area of interest. In the literature, very few dedicated research support tools are available as per the authors understanding. The closest works to this study can be found in Simon et al. (1). Here the authors have presented a text mining based tool called BioReader for the classification of Biomedical research. In (2), R-classify is a web tool developed by Aggarwal et al. to help users in finding out the relevant literature in the area of Computer Science. Doty et al. (3) developed a python-based graphical user interface to conduct the classification and visualization of electron microscopy data.

In the present article, an Artificial Intelligence (AI) enabled automatic classification tool called Research Support Tool (RST) is developed for COVID-19-related literature. Since the problem is of text (literature) classification, a Bi-LSTM neural network is used. The Bi-LSTM model is trained on the abstract and title of the selected articles. The articles are taken from the CORD-19 dataset and are divided into seven categories (class labels) based on their subjects. The RST is developed using IONIC and Angular framework. Remaining of the article consists of three more sections. In Section 2, the methodology followed in the present study is described. In Section 3, the user interface is presented, and the workflow is defined. Finally, Section 4 provides the concluding remarks and also provides some future directions in which the work can be extended.

## 2. Materials and methodology

**Figure 5:**
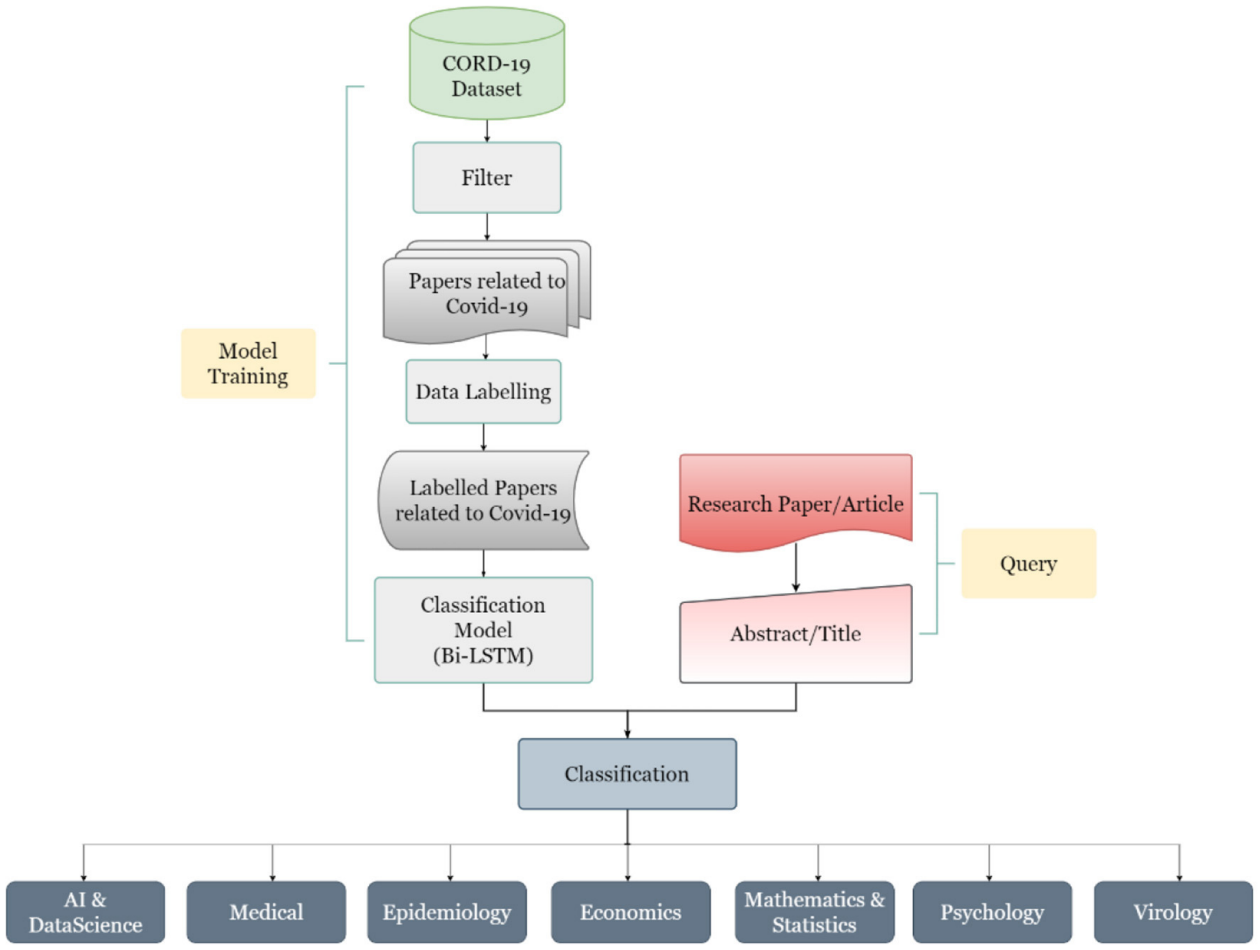
The workflow diagram of the AI-enabled research support tool process.

The work done in this study can be divided into four major steps, which start from data collection to its preparation to its labeling and finally to its classification. The steps are defined below in Figure 5.

### 2.1. Step 1: Data collection

The first step in this study is the collection of data for which the COVID-19 Open Research Dataset or CORD-19 (4) was selected. It is curated by Allen Institute for AI (AI2) and is available on Kaggle (5) as well as on AI2's Semantic Scholar website (6). This database is periodically updated. At the time of the preparation of this article, it contained resources for almost 4,00,000 scholarly articles, including over 2,45,000 full-text articles on COVID-19, SARS-CoV-2, and variants of coronaviruses.

### 2.2. Step 2: Data preparation

Once the data source has been identified, the next step is to prepare the data for further usage. To make the study more relevant in terms of the COVID scenario, only the studies subjected to COVID-19 or SARS-CoV-2 were considered. This was done by using the keywords like “COVID-19,” “Wuhan,” “Hubei,” “SARS-CoV-2,” “2019 novel coronavirus,” “2019-nCoV,” “coronavirus disease 2019,” “corona pandemic,” “coronavirus outbreak,” and their combinations and filtering out the studies not meeting up with our criteria. Initially, 4,532 articles were selected based on different subjects, after filtering out the inconsistent, incomplete data, a total of 3,011 articles are taken for the model training and database development.

### 2.3. Step 3: Data labeling

**Figure 6:**
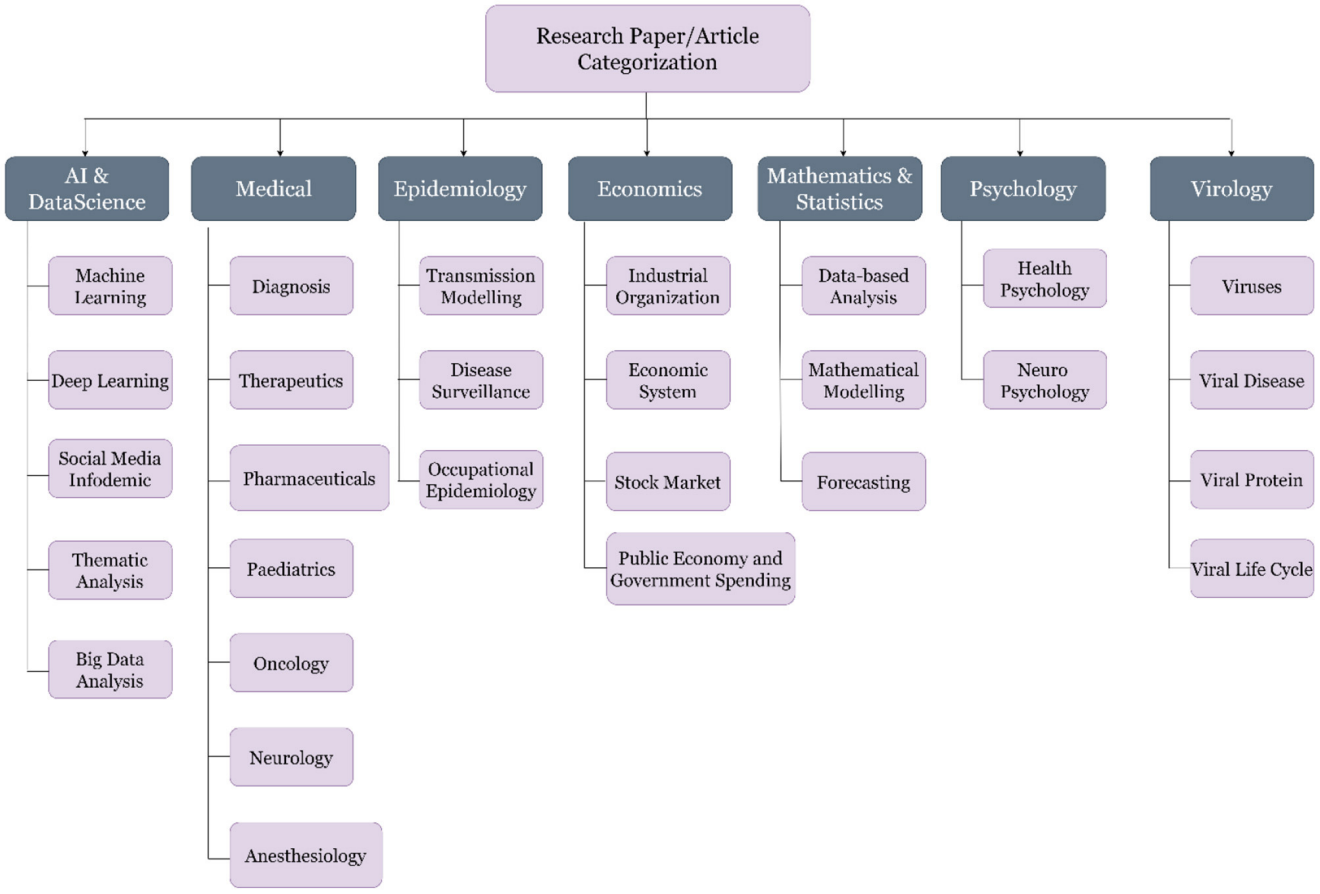
Categorization and sub-categorization of the articles selected for the study.

The third step, and also one of the key tasks of this study, was to label the articles, which can be classified later as per the machine learning algorithms. The literature was segregated into seven major classes per the experts' discussion. These seven classes are Artificial Intelligence (AI) and Data Science, Economics, Epidemiology, Mathematics and Statistics, Medical, Psychology and Virology. A brief description of the classes is shown in Table 1, and the subclasses of the selected articles are shown in Figure 6.

**Table 1:** A description of class labels categorization.

**Class**	**Description**	**References**
Artificial Intelligence (AI) and Data Science	This class is divided into five subclasses of AI and Data Science—machine learning, deeplearning, social media infodemic, thematics analysis, and big data analysis for selecting the related articles	(7–15)
	The articles that deal with AI and Data Science being used for automatic screening of COVID-19 using computer tomography scans and X-ray images of the lungs of patients, prediction and forecasting of virus spread, mortality risk etc. It is further subdivided into Machine Learning and Deep Learning, Data Mining, Data Analysis methods for social media infodemic, misinformation spreading, patient report analysis, sentiment analysis, infoveillance, and information on datasets which are relevant to deal with COVID-19 are classified under this category	
Economics	This class has four subcategories—industrial organization, economic system, stock market, public economy and government spending. The articles that belong to these categories discuss the consequences of COVID-19 on the economy of a country, the economy of a specific product, the economy of a segment of the market, and stock markets are classified under this category	(16–20)
Epidemiology	This class considers three subcategories—transmission modeling, disease surveillance, and occupational epidemiology. The articles categorized in this class deal with outbreak control measures, the effect of COVID-19 on various occupations and environments, risk assessment, transmission monitoring, transmission pattern recognition, analysis and forecasting are classified under this category	(21–25)
Mathematics and Statistics	This class considers three subcategories—data-based analysis, mathematical modeling, and forecasting. Articles that explain how mathematical modeling and statistical analysis are utilized to predict the transmission and spread of COVID-19 and also to identify the mitigation strategies are classified under this category	(26–30)
Medical	Diagnosis, therapeutics, pharmaceuticals, pediatrics, oncology, neurology, and anesthesiology are the subclasses of class medical. The papers dealing with COVID-19 diagnosis, therapeutics, immunology, pharmacology, anaesthesiology, oncology, neurology, pediatrics, hematology etc. medical related issues are classified under this category	(31–34)
Psychology	Two subcategories—health psychology, and neuropsychology are considered for selecting the articles that belong to this class. The papers that discuss the impact of the COVID-19 epidemic on the mental health and psyche of human beings and their behavior are classified under this category	(35–39)
Virology	There are four different subclasses—viruses, viral disease, viral protein, and viral life cycle are considered for this class. The papers with research work on the virus structure, genome, molecular characterization, and mutation are classified under this category	(40–42)

**Figure 7:**
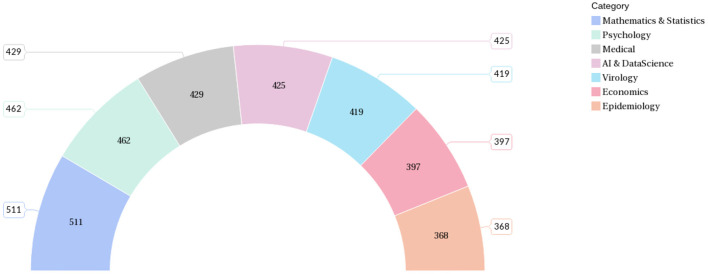
Number of data samples (articles) assigned to the class categories.

In the dataset created for this work, each data contains the title, abstract, and class label of the literature. The data distribution among the selected seven categories is shown in Figure 7.

### 2.4. Step 3: Classification

The AIRST developed in the present study is based on the classification of text, for which the Bi-directional long short-term memory (Bi-LSTM) neural network (43) is implemented. Vanilla neural networks are not found to be suitable for texts as these are unable to process the sequences.

Recurrent neural networks (RNN), have a loop-like architecture which allows the information to persist. RNNs have been successfully applied to various areas including speech recognition, speech synthesis, language translation, image captioning and many more (44–46). However, in the case of sequential data, it sometimes becomes susceptible to vanishing gradient due to long-term dependency. The problem of vanishing gradient can be resolved with the help of LSTMs (47), a type of RNN which are capable of learning long-term dependencies. The LSTM models are made up of cell states and various gates. While the cell state in LSTM acts like a memory of the network and transfers relevant information down the sequence chain model; gates are the neural networks that decide the information to be retained and the information to be forgotten during training. An LSTM model consists of three gates viz. forget gate, input gate, and output gate. These gates are described in brief as follows.

#### 2.4.1. Forget gate

The first step of the LSTM cell is to retain the relevant information and to discard the information that is not of significance. This is done with the help of the sigmoid layer known as the “*forget gate layer.”* The activation value for the forget gate can be given as:


(1) $$\begin{array}{l} f_{t}=\sigma \left(w_{f}\left[h_{t-1},x_{t}\right]+b_{f}\right) \end{array}$$

where *x*_*t*_ is input vector at timestamp *t h*_*t*−1_is a hidden state or output of the previous timestamp, *w, b* represent the weight and deviation matrix, respectively.

The sigmoid function normalizes all the activation values between 0 and 1. The value 0 implies all forgotten, and the value 1 implies nothing forgotten.

#### 2.4.2. Input gate

The second step in an LSTM model is to identify the information that will be stored in the state of a cell. The input gate layer quantifies the crucial information carried by the input. This step is further divided into two parts. First, an “input gate layer” (sigmoid layer) decides the values to be added to the cell state *C*_*t*_ and then, a tan*h* layer derives a vector of new candidate value *N*_*t*_, that has to be added to the state. This is followed by the combination of the aforementioned steps to update the state. The input gate activation value is as follows:


(2) $$\begin{array}{l} i_{t}=\sigma \left(w_{i}\left[h_{t-1},x_{t}\right]+b_{i}\right) \end{array}$$

where, *x*_*t*_ is input vector at timestamp *t*, *h*_*t*−1_is a hidden state or output of the previous timestamp, *w, b* represent the weight and deviation matrix, respectively.

*N*_*t*_ is defined as:


(3) $$\begin{array}{l} N_{t}=\tanh\left(w_{c}\left[h_{t-1},x_{t}\right]+b_{C}\right) \end{array}$$

Cell state is updated as:


(4) $$\begin{array}{l} C_{t}=f_{t}^{*}C_{t-1}+i_{t}^{*}N_{t} \end{array}$$

Where, *C*_*t*−1_ is the previous cell state.

#### 2.4.3. Output gate

The objective of the output gate is to decide the output which in turn will be *n* the basis of the state of the cell. Here, a sigmoid layer identifies the part of the cell state that will be the output. This information is further processed by passing the cell state through the activation function tan*h* and multiplying it with the output of the sigmoid gate. Finally, the output *h*_*t*_ is obtained as:


(5) $$\begin{array}{l} O_{t}=\sigma \left(w_{o}\left[h_{t-1},x_{t}\right]+b_{o}\right)h_{t}=O_{t}^{*}\tanh\left(C_{t}\right) \end{array}$$

#### 2.4.4. Bi-directional long short-term memory

**Figure 8:**
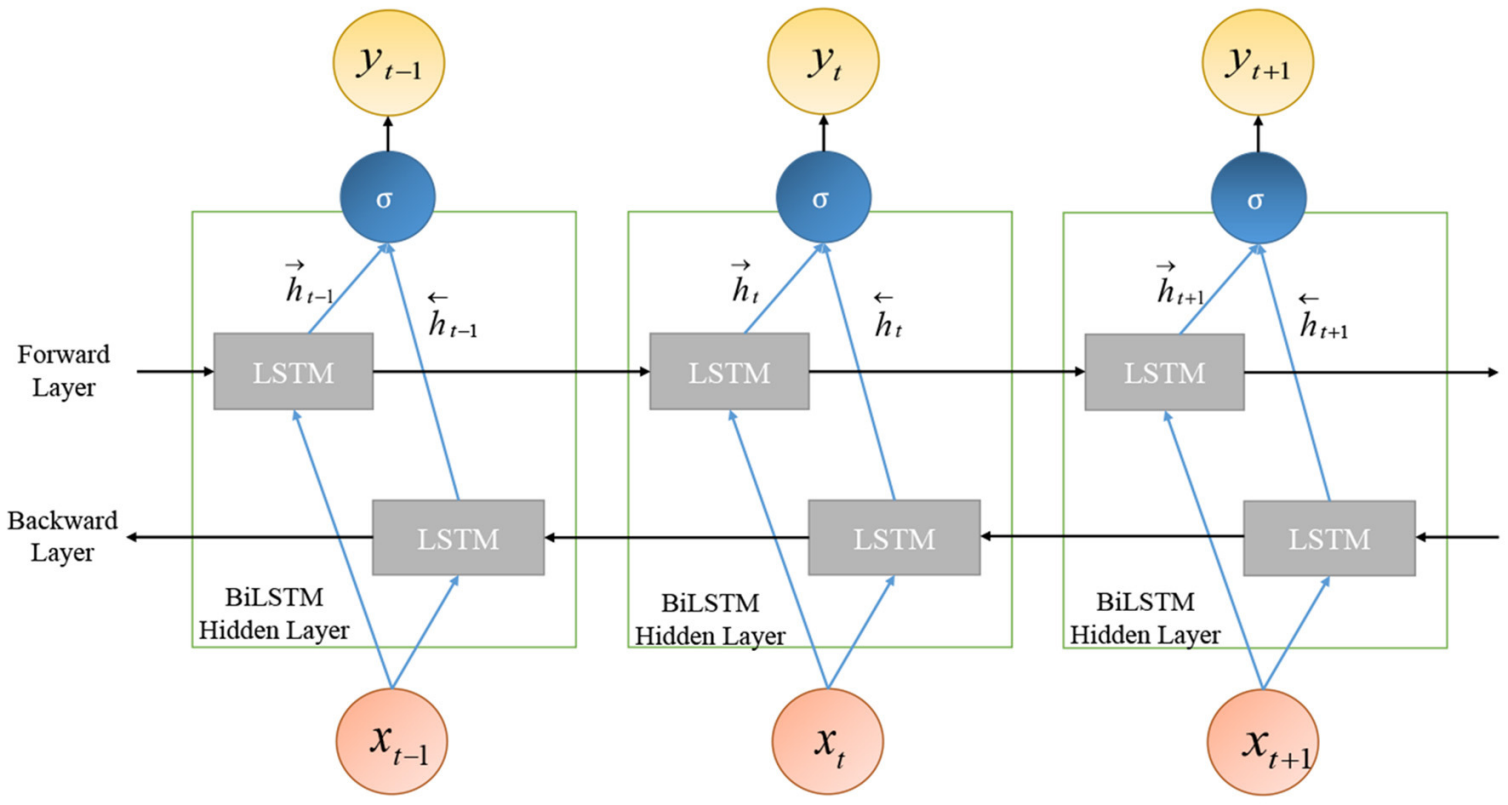
Bi-directional long short-term memory (Bi-LSTM).

Bi-directional long short-term memory (Bi-LSTM) is an extended and improved version of LSTM; it is an integration of two independent RNN models. Unlike unidirectional LSTM, in Bi-LSTM, the information flows in both directions: backward as well as in the forward direction. This is illustrated in Figure 8.

Bi-LSTM exploits the information about the sequence in both directions at every timestamp by connecting two hidden layers to the same output. The management of the past and future information, for a sequence, leads to better predictions for Bi-LSTM. The output of the hidden layer of Bi-LSTM is made up of the activation output of forward as well as backward hidden layers:


(6) $$\begin{array}{l} {\overset{\to }{h}}_{t}=\sigma \left(W_{x\overset{\to }{h}}x_{t}+W_{\overset{\to }{h}\overset{\to }{h}}\overset{\to }{h_{t-1}}+b_{\overset{\to }{h}}\right) \end{array}$$

(7) $$\begin{array}{l} {\overset{\leftarrow }{h}}_{t}=\sigma \left(W_{x\overset{\leftarrow }{h}}x_{t}+W_{\overset{\leftarrow }{h}\overset{\leftarrow }{h}}\overset{\leftarrow }{h_{t-1}}+b_{\overset{\leftarrow }{h}}\right) \end{array}$$

(8) $$\begin{array}{l} H_{t}=W_{x\overset{\to }{h}}\overset{\to }{h}+W_{\overset{\leftarrow }{h}y}\overset{\leftarrow }{h}+b_{y} \end{array}$$

where, *H*_*t*_ represents the hidden layer, and its output includes the forward layer output ${\overset{\to }{h}}_{t}$ and backward layer output ${\overset{\leftarrow }{h}}_{t}$.

The Bi-LSTM model was trained on a total of 3,011 samples of seven different categories of research articles related to COVID-19 that are collected from the CORD-19 dataset. The parameters of the Bi-LSTM model architecture are mentioned in Table 2.

**Table 2:** Parameters of Bi-LSTM model architecture.

**Parameters**	**Size**
Embedding layer vocab size	10,000
Embedding dimension	64
Maximum length of a unique word	200
Bi-LSTIM size	32
Batch size	64

The final layer of the model is the Dense output layer with seven neurons representing the total number of class labels and Softmax activation function. To avoid overfitting while training the model, each layer is followed by the Dropout layer with an alpha value as 0.35.

### 2.5. User interface and workflow

The workflow of the research support tool has two components—the objective of the user interface development and the cloud environment-based application development tools.

#### 2.5.1. Objective

A research support tool has been designed to meet the following three primary objectives:

1. Enable users to view COVID-19-related research papers and articles under different categories. The users are also enabled to filter and search for research papers based on the title of the research papers.
2. Enable users to categorize an article not available in the dataset. The user can do that by providing DOI and proceeding after checking the extracted title and abstract.
3. Enable users to contribute to the labeled dataset by providing the title and abstract of the research paper and assigning a category manually.

#### 2.5.2. Application development tool

A cloud environment-based application was developed that used a micro-service architecture to meet the mentioned requirements. The following technology stack was selected to develop the tool:

1. Azure Cloud platform—Azure Cosmos DB (NoSQL) and Azure Cloud Functions were used for storing and retrieving data, executing the Python script to categorize research papers based on the trained model.
2. Ionic + Angular—Ionic and Angular frameworks were used to develop the user interface because of easily available components and ability to deploy on multiple platforms such as Desktop, Mobile (Android and iOS using Cordova or Capacitor), Progressive Web Apps (PWA) and Cloud Hosted Web.
3. NodeJ—NodeJS middleware was used to access micro-services and respond to user interactions.

The workflow of the developed user interface consists of three parts: (1) use of helper APIs, (2) load data for training and training the model, (3) evaluation: evaluation again consists of two parts—the use of helper APIs and Evaluation against the model. The complete process of user interface workflow is shown in Table 3.

**Table 3:** A detailed workflow of the developed user interface.

*Use of helper APIs* 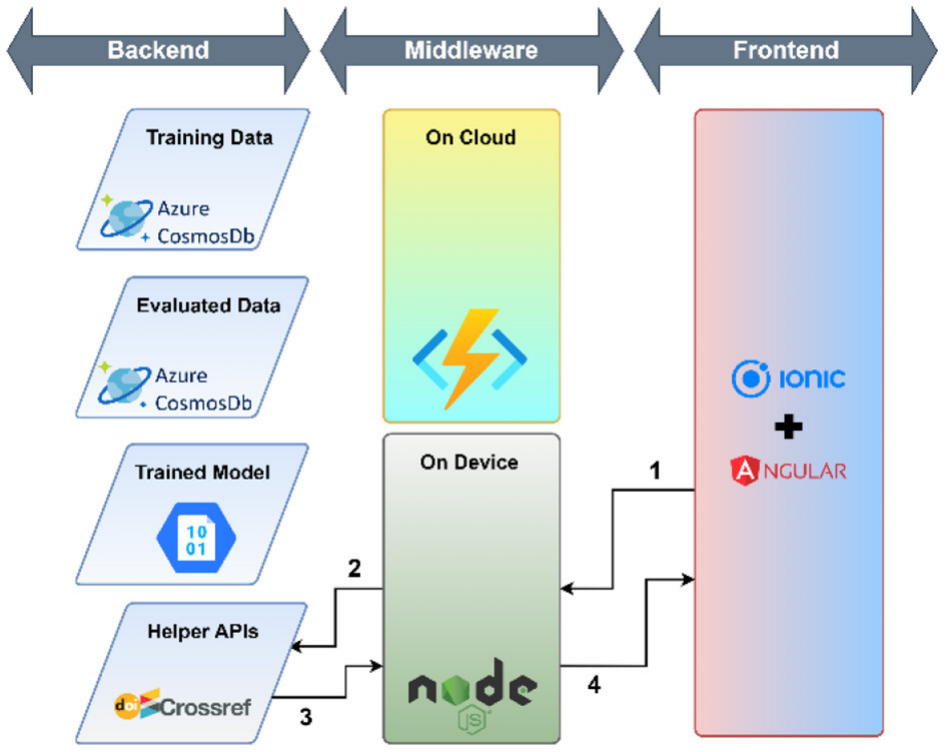	The application utilizes DOI to ensure the uniqueness of documents in the training and evaluation dataset. CrossRef APIs are used to make the user experience smoother for the end-user. Since the CrossRef API does not guarantee that the abstract will be available in all DOIs, or the veracity of the abstract, the end-user can make adjustments to the abstract to ensure it is correct. The following steps are executed— 1. The user enters the DOI in the textbox provided. Once the user clicks on the check button, the NodeJS service picks the DOI 2. The DOI is passed the CrossRef Works API 3. The CrossRef database returns the details of the work in a semi-structured JSON 4. The Title and Abstract from the response are extracted and displayed to the end user
*Load data for Training* 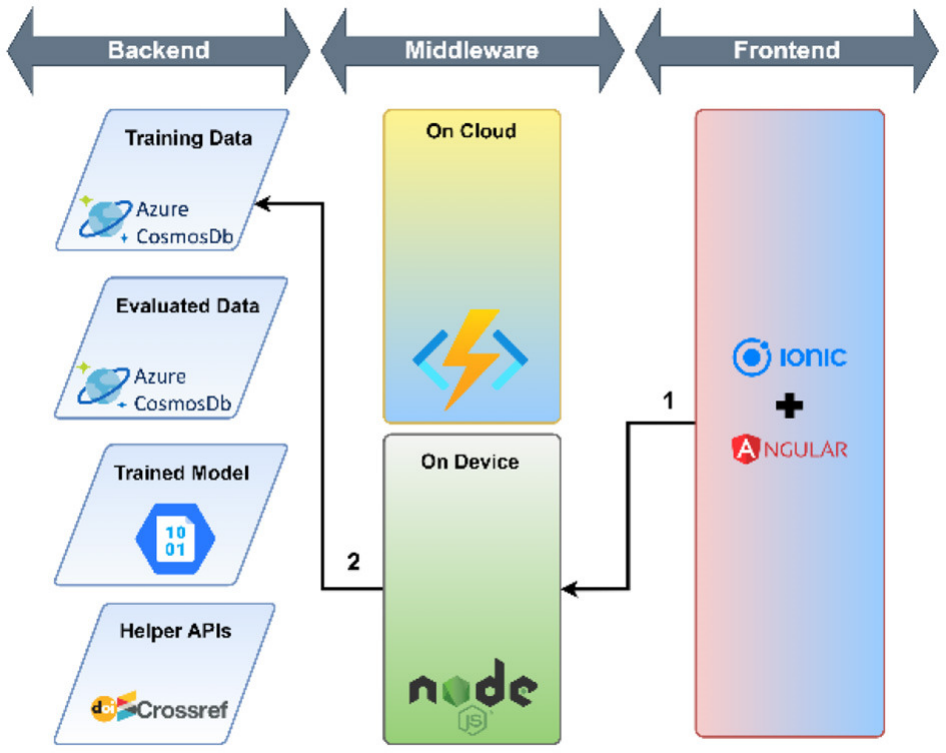	Once the user has entered the DOI, the title and abstract have been verified, the user can select the category. The following steps are executed— 1. The user ensures the title and the abstract are correct and fall under the selected category from the drop-down. When the user clicks on the “Submit for Training” button the details are passed to the NodeJS Service 2. The NodeJS service ensures that the data provided is in the correct structure. If the same DOI is present in the training dataset, the service will overwrite the record. Otherwise, the service will create a new record in the training dataset
*Model Training* 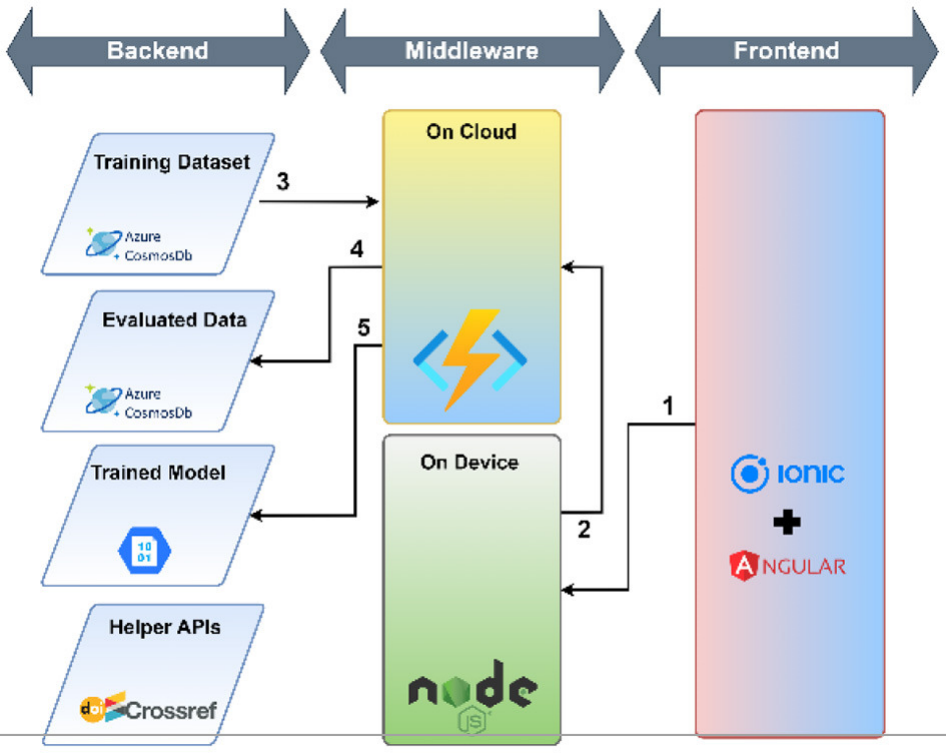	The following steps are executed— 1. The user clicks on the “Retrain Model” button on the “Submit New” page which triggers the NodeJS service 2. The NodeJS service authenticates and triggers an Azure Cloud Function to retrain the model 3. The Azure Cloud Function fetches all the records from the training dataset 4. The Azure Cloud Function deletes all the records from the evaluation dataset 5. The Azure Cloud Function converts the training dataset in the required format and trains the model and stores it in the Azure Blob Storage service
*Evaluation against the model* 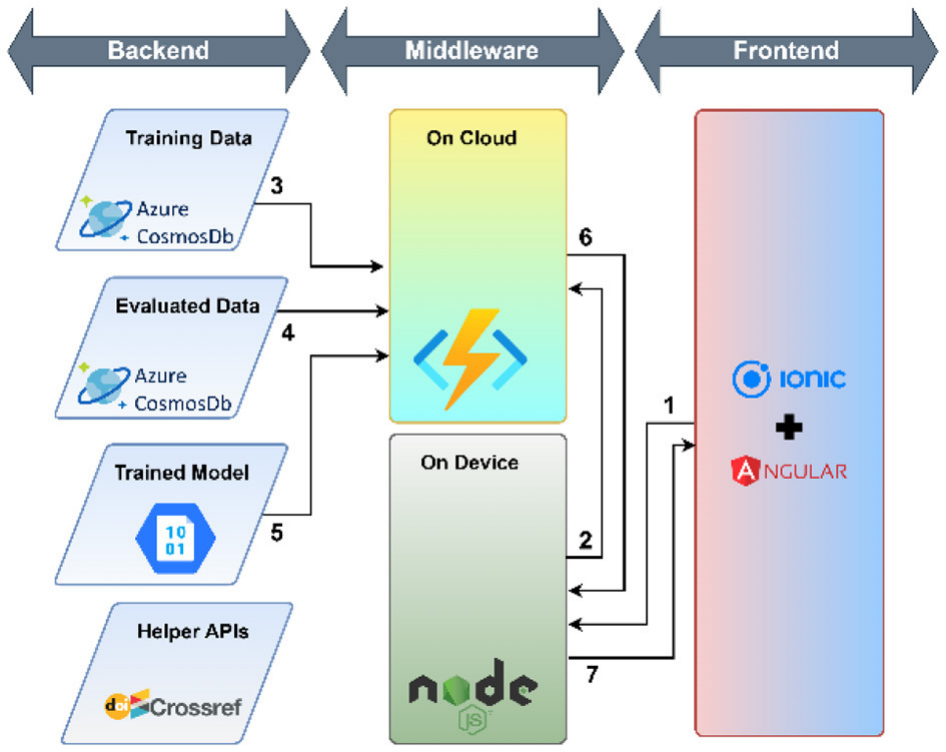	The following steps are executed 1. Once the user ensures that the DOI, Title and Abstract are correct and clicks on the “Get Category” button, the details are passed to the NodeJS Service 2. The NodeJS service authenticates with the Azure Cloud function and passes the DOI, Title and Abstract as arguments to the Azure Cloud Function 3. The Azure Cloud Function checks whether the DOI is present in the training dataset. If the DOI is present in the Training Dataset, the cloud function will return the category present in the training dataset 4. The Azure Cloud Function checks whether the DOI is present in the evaluation dataset. If the DOI is present in the Evaluation Dataset, the cloud function will return the category present in the Evaluation Dataset 5. Steps 3 and 4 are done to reduce unnecessary computation against the model since it is a computationally expensive process. If the DOI is not present in either the training or evaluation dataset, the Azure Cloud Function will retrieve the model stored in the Azure Blob Storage and evaluate the category against the provided Title and Abstract 6. The Azure Cloud Function returns the evaluated Category to the NodeJS service and stores the DOI, Title, and Abstract along with the category in the Evaluation Dataset 7. The NodeJS service displays the evaluated Category against the given DOI, Title and Abstract

## 3. Results and analysis

**Figure 9:**
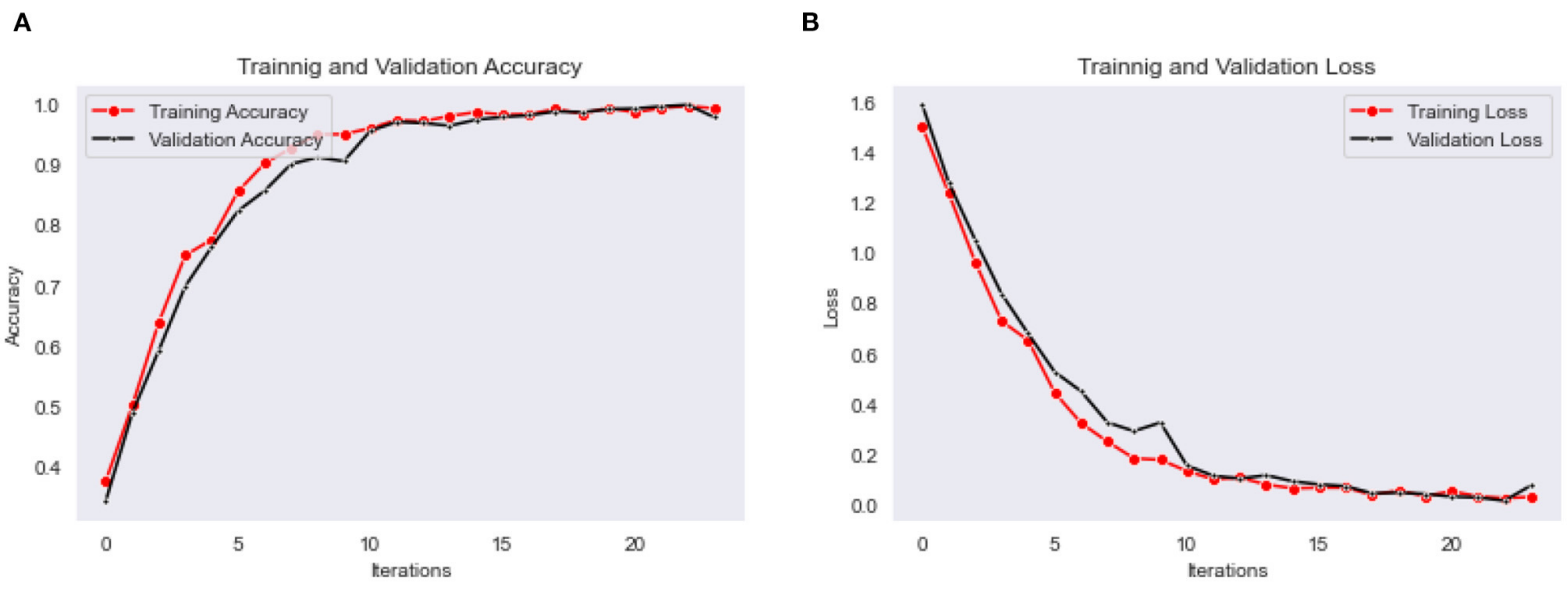
**(A)** Training and validation accuracy, **(B)** training and validation loss plots of Bi-LSTM classification model.

The Bi-LSTM classification model is trained for the 25 epochs, and obtained maximum validation accuracy as 0.97, with a minimum validation loss as 0.015. The accuracy and loss for every epoch of training and validation are shown in graphs plots in Figures 9A, B, respectively.

The performance of the research support tool is presented through the screen captures of the developed user interface. Users can see the following view upon landing. The view is divided into three segments to meet the three objectives mentioned above. These segments can be accessed using the three tabs at the bottom of the interface.

**Figure 10:**
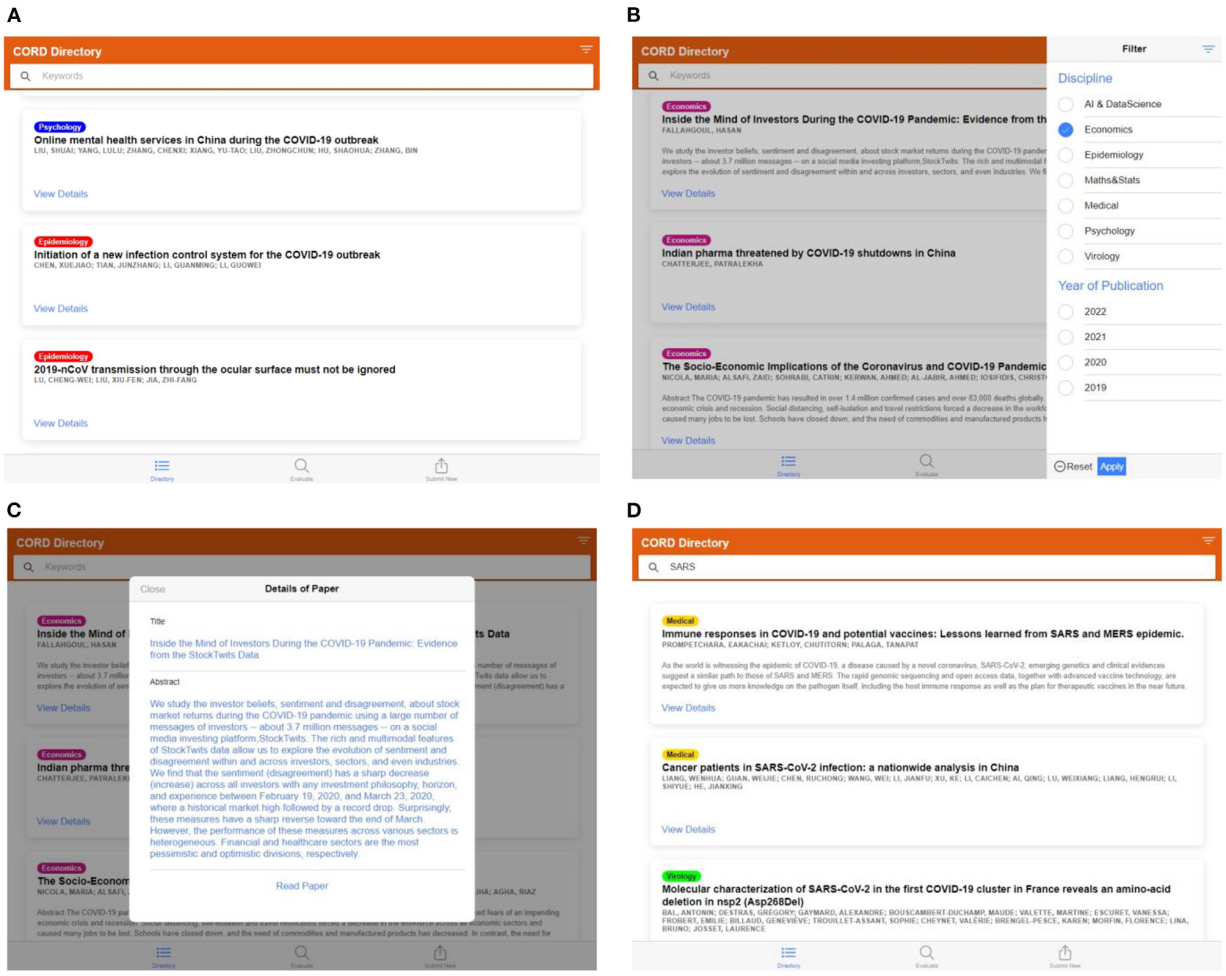
**(A)** Screen capture of the “Directory” tab—list of labeled articles in the database, **(B)** filter panel on the left side to select the articles of the particular category, **(C)** details of the listed articles, read paper tab will redirect to the original article page through DOI, **(D)** keyword tab can be used to search the article from the labeled dataset.

**Figure 11:**
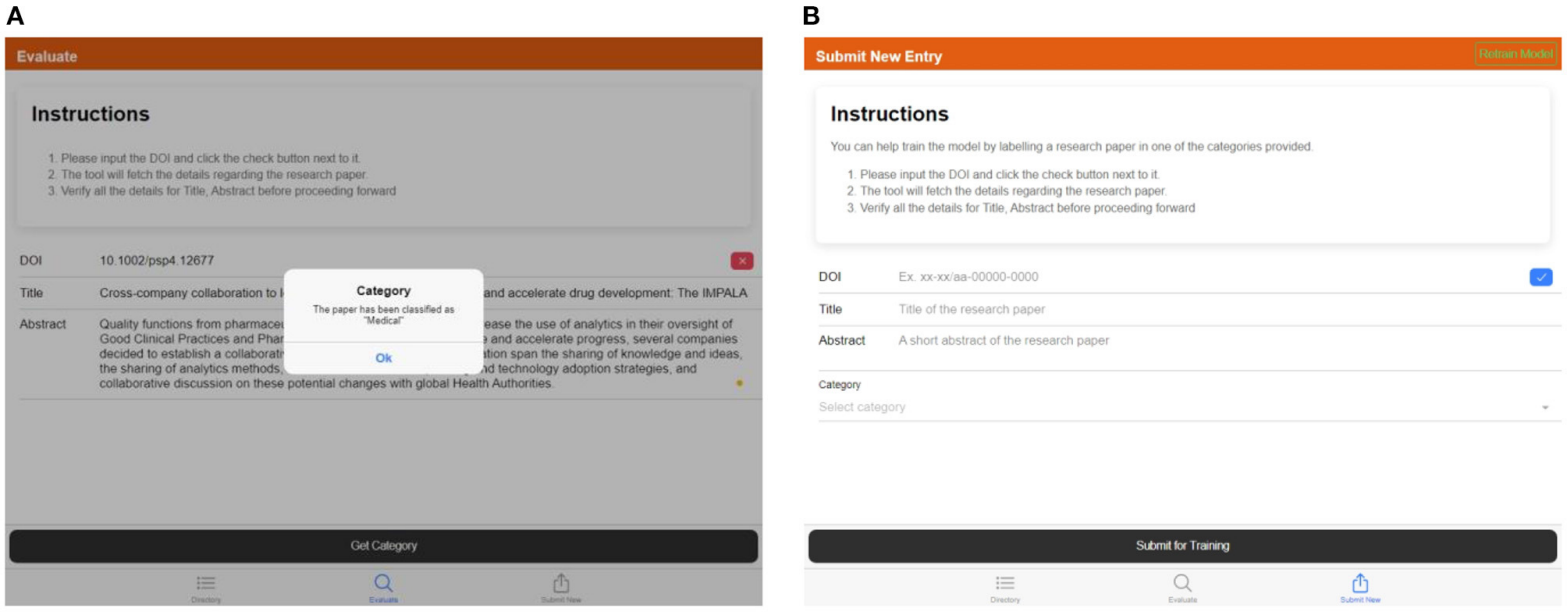
**(A)** Screen capture of the “Evaluate” tab—popup showing the category of the provided DOI, **(B)** screen capture of the “Submit New Entry” tab—details of the article can be provided by manually feeding the DOI, title, abstract, and the relevant category.

1. The “Directory” tab is used to view, search and filter the research papers already categorized by the model. These include records from the training dataset and any records generated when a customer is evaluating a research paper using the model, shown in Figures 10A–D.
2. The “Evaluate” tab is used to provide the details of a research paper and categorize it using the trained model, shown in Figure 11A.
3. The “Submit New Entry” tab is used to manually label any research paper and add it to the training dataset. This will allow us to grow the training dataset and re-train the model periodically, as shown in Figure 11B.

The user interface requests DOI to enable CrossRef API to get details regarding the research paper, such as the title and abstract.

## 4. Conclusion and future scope

This work primarily intends to communicate the idea of developing a Research Support Tool for researchers around the world. The conclusive statements can be drawn from this study as shown below:

- The researchers can leverage this tool to delve deeper into COVID-19 research and make the relevant literature identification smoother.
- A multi-platform graphical user interface is developed to fulfill the primary objectives of extracting the COVID-19 related articles effortlessly and classifying them based on the particular research area.
- The classification system uses the Bi-LSTM model, which enhances efficiency by feeding the input in both backward and forward directions. The results regarding the system's performance have been presented.
- The research support tool can further be extended for different research areas, and the classification model can also be trained on different datasets for other application areas.
- This article considers the abstract and title while training the model. In future, the conclusion and the related work part of the articles can also be included for increasing the better exploration.

## Data availability statement

The original contributions presented in the study are included in the article/supplementary material, further inquiries can be directed to the corresponding authors.

## Author contributions

KB: interpretation and acquisition of data. AT: development of model architecture and user interface. MP, SS, and VS: conception of ideas and formulation and development of designing concepts. All authors contributed to the article and approved the submitted version.
